# Comparison of Non-invasive Diagnostic Methods for *Helicobacter Pylori* Infection Before and After Treatment

**DOI:** 10.34172/mejdd.3286

**Published:** 2026-01-31

**Authors:** Seyedeh Azra Shamsdin, Hossein Faramarzi, Hajar Khazraei, Fateme Zare, Amirhossein Kamyab

**Affiliations:** ^1^Gastroenterohepatology Research Center, Shiraz University of Medical Sciences, Shiraz, Iran; ^2^Department of Community Medicine, School of Medicine, Shiraz University of Medical Sciences, Shiraz, Iran; ^3^Colorectal Research Center, Shiraz University of Medical Sciences, Shiraz, Iran; ^4^Student Research Committee, Shiraz University of Medical Sciences, Shiraz, Iran; ^5^Faculty of Medicine, Fasa University of Medical Sciences, Fasa, Iran

**Keywords:** *Helicobacter pylori*, IgG, Stool antigen, Infection, Treatment

## Abstract

**Introduction:**

*Helicobacter pylori* (*H. pylori*) infection is a major contributor to peptic ulcer disease and other gastrointestinal disorders. The present study aimed to investigate the effect of treatment on diagnostic outcomes by comparing two non-invasive methods—the stool antigen test and the serological IgG antibody test, before and after therapy. This approach was designed to evaluate not only the diagnostic efficacy of these tools but also the changes in antibody responses following eradication treatment.

**Methods:**

This observational study was conducted from 2021 to 2022 at Shahid Motahari Clinic, affiliated with Shiraz University of Medical Sciences, Shiraz, Iran. A total of 108 patients with dyspepsia and confirmed *H. pylori* infection were enrolled. Diagnostic tests included stool antigen, anti-*H. pylori* IgG serology, and the urease breath test (UBT). Patients received standard triple therapy, with stool antigen and IgG levels measured before and one month after treatment to assess diagnostic accuracy and treatment outcomes.

**Results:**

Among 108 patients, 74% had peptic ulcer disease. The UBT showed the highest diagnostic accuracy (100% sensitivity, 94.6% specificity). Anti-*H. pylori* IgG serology had high sensitivity (100%) but lower specificity (66.2%). The stool antigen test demonstrated moderate accuracy. After treatment, stool antigen levels significantly decreased (from 4.2±0.64 to 2.9±0.75, *P*=0.03), and IgG titers declined significantly (from 54.26±3.33 to 46.51±3.22, *P*<0.0001).

**Conclusion:**

Our findings underscore UBT as the most accurate method and highlight the value of stool antigen testing for follow-up. Monitoring IgG titer changes may support treatment assessment when used alongside other tools, though its low specificity limits stand-alone use.

## Introduction

*Helicobacter pylori* (*H. pylori*) is one of the most prevalent bacterial infections in humans, and its discovery revolutionized the diagnosis and treatment of gastric and duodenal diseases.^1^ It is considered the world’s most common chronic infection, with a global prevalence of 44.3%-34% in developed countries and 51% in developing ones^2,3^ Higher prevalence rates are reported in regions such as Russia, Iran, China, and Latin America, whereas lower rates are observed in the Netherlands and Oceania.^4^

 Infection usually occurs in early childhood and is mainly transmitted person-to-person, often within families or in settings with poor hygiene. In developing countries, environmental factors such as contaminated food and water further contribute to endemic infection.^5-7^ *H. pylori* is not only a major cause of peptic ulcer disease but also a well-established risk factor for gastric adenocarcinoma and MALT lymphoma.^8,9^ Moreover, studies suggest associations with extra-gastrointestinal conditions, including neurological, hematological, metabolic, and cardiovascular disorders.^10,11^

 Several diagnostic methods are available for identifying *H. pylori* infection, with the choice depending on factors such as availability, advantages and disadvantages, cost, and the patient’s age. The urea breath test (UBT) is a common method that detects labeled carbon in exhaled air, though it requires special equipment and is costly.^12^ The stool antigen test is another frequently used non-invasive method. Serological tests, such as anti-*H. pylori* IgG and IgM measurements are also employed.^13^ Studies have compared the accuracy of these diagnostic tools. For example, one study of 100 Iranian students with dyspepsia reported that the stool antigen test had a positive predictive value of 85.42% and a negative predictive value of 80.77%, supporting its role as a reliable test for diagnosis and follow-up.^14^ Similarly, a study on 806 Japanese high school students found that the urine antibody test demonstrated a sensitivity of 97.6%, a specificity of 96.5%, a positive predictive value of 61.2%, and a negative predictive value of 99.9%, highlighting its potential as an effective and easy-to-use screening tool.^15^ Another investigation, using histopathological testing of gastric mucosa as the gold standard, confirmed the stool antigen test’s diagnostic value, with a sensitivity of 80.39%, a positive predictive value of 85.42%, and a negative predictive value of 80.77%.^16^

 Even with these improvements, serological tests often yield positive results in uninfected individuals in regions with high prevalence, and no universally accepted cut-off exists to distinguish active from past infection. This, combined with the limitations and errors of other diagnostic methods, underscores the need to examine and compare antibody titers before and after treatment. Such an approach may clarify whether quantitative IgG levels can serve as a reliable criterion for differentiating active from previous *H. pylori* infection. Since current diagnostic methods have notable shortcomings and no single test provides perfect accuracy, this study was designed to evaluate changes in anti*-H. pylori* IgG levels and stool antigen test results before and after treatment. The findings may improve diagnostic precision, offer more practical follow-up strategies, and ultimately contribute to better patient management in populations where *H. pylori* is highly prevalent.

## Materials and Methods

###  Study Environment and Sample Size

 This observational case-control study was conducted between January 2021 and December 2022 at Shahid Motahari Clinic, affiliated with Shiraz University of Medical Sciences, Shiraz, Iran. The study aimed to compare two non-invasive diagnostic methods, stool antigen testing and anti-*H. pylori* IgG serological testing, before and after treatment for *H. pylori* infection. Initially, 135 patients presenting with dyspeptic symptoms were recruited. After excluding patients for non-compliance with medication or follow-up, the final sample size consisted of 108 patients. The sample size was calculated using a formula with 90% power and an alpha level of 0.05 to ensure adequate statistical significance for comparing proportions.

###  Inclusion and Exclusion Criteria

 Adults over 18 years old with dyspeptic symptoms and confirmed *H. pylori* infection were included in the study. Confirmation of infection was based on invasive diagnostic methods. During endoscopy, gastric mucosal biopsy samples were obtained from the antrum and corpus. The rapid urease test (RUT, CLO test) was performed by placing biopsy specimens into urea-containing medium with a pH indicator; a color change from yellow to red within 24 hours was considered positive. For histopathological examination, biopsy samples were fixed in formalin, embedded in paraffin, and stained with hematoxylin–eosin and Giemsa to detect *H. pylori* organisms and assess gastritis. Endoscopic findings consistent with gastritis or ulcer disease, in combination with a positive RUT or histology, were used as diagnostic confirmation.

 Patients with compromised immune systems, those undergoing immunosuppressive therapy, or those who had used proton pump inhibitors (PPIs) or antibiotics within two weeks before testing were excluded to avoid diagnostic interference.

###  Data Collection

 Patients with dyspepsia symptoms and confirmed *H. pylori* infection, based on positive stool antigen, rapid urease test (CLO test), pathology results, or UBT, were recruited from the gastroenterology or infectious disease clinics of Motahari Clinic. Inclusion criteria were applied to patients who provided informed consent and met the requirements, while those who met the exclusion criteria were excluded from the study.

 A comprehensive questionnaire was administered at the beginning of the study and again one month after the completion of drug treatment, when the second stool antigen test was conducted. Information collected included patient demographics, medical history (with emphasis on congenital or acquired immune deficiencies), and medication history (with emphasis on immunosuppressive drugs, antibiotics, and PPIs). Gastrointestinal symptoms, duration of symptomatic periods, history of endoscopy, UBT results, and stool antigen test findings were recorded.

 The UBT was performed by having patients ingest a 75 mg ^13C-urea solution after an overnight fast. Breath samples were collected before ingestion and 30 minutes afterward. The presence of *H. pylori* urease activity was determined by measuring the change in ^13CO₂/^12CO₂ ratio in exhaled air using an infrared spectrophotometer. A significant increase in ^13CO₂ indicated a positive result.

 Medication adherence and compliance with the 1-month interval between completing drug treatment and stool antigen testing were evaluated in the follow-up questionnaire. Patients who did not adhere to these requirements were excluded from the study.

###  Treatment Steps

 After obtaining consent and completing the questionnaire, a 5-cc blood sample was collected for serology testing, along with a quantitative stool antigen test result. A 14-day treatment regimen consisting of clarithromycin (500 mg every 12 hours), amoxicillin (1 g every 12 hours), and pantoprazole (40 mg every 12 hours) was initiated. Instructions on the importance of regular medication use and the necessity of discontinuation upon completion were provided. One month after the drug treatment ended, patients were asked to return for a follow-up stool antigen test and serology test to assess treatment outcomes. A one-month interval was maintained to prevent false-negative stool antigen test results.

###  Stool Antigen Test Procedure for H. Pylori

 Each patient provided a fresh stool sample in a sterile container, which was transported to the laboratory under cold conditions. Quantitative stool antigen levels were determined using a commercial ELISA kit (MONOBIND, USA).^17^ The assay was performed according to the manufacturer’s instructions: stool suspensions were prepared, loaded into ELISA wells along with calibrators and controls, incubated, washed, and developed with chromogenic reagents. Optical density was measured at 450 nm, and quantitative results were obtained using the kit’s standard curve. This method has been validated as a reliable, non-invasive tool for the diagnosis and follow-up of *H. pylori* infection

###  Anti-H. Pylori IgG Test Procedure

 Venous blood samples (5 cc) were obtained from each patient, centrifuged, and the sera were stored at −20 °C until analysis. Anti-*H. pylori* IgG titers were measured using the MONOBIND ELISA kit (USA).^17^ Following the manufacturer’s protocol, diluted serum samples, controls, and standards were added to pre-coated wells, incubated, washed, and incubated with enzyme conjugate. After the addition of the substrate and stop solution, absorbance was measured at 450 nm, and antibody concentrations were calculated from the standard curve. ELISA-based IgG testing is a widely used serological method for evaluating exposure and response to *H. pylori* infection.

###  Statistical Analysis

 Data were analyzed using SPSS software, version 26.0 (IBM Corp., Armonk, NY, USA). The Shapiro-Wilk test was used to assess the normality of the data distribution. The Generalized Estimating Equation (GEE) model was applied to compare the two tests (*H. pylori* stool antigen test and anti-*H. pylori* IgG antibody test) before and after treatment. The comparison between tests was performed using the *t* test, and a P value of less than 0.05 was considered statistically significant.

###  Ethical Considerations

 This study was conducted in accordance with the ethical standards of the Declaration of Helsinki and the International Committee of Medical Journal Editors (ICMJE) guidelines for biomedical research involving human subjects. Ethical approval was obtained from the Ethics Committee of Shiraz University of Medical Sciences, Shiraz, Iran (Approval No: IR.SUMS.MED.REC.1400.122). All participants provided written informed consent after receiving a full explanation of the study objectives, procedures, and their rights, including the right to withdraw at any time without consequence. Confidentiality and privacy of participants’ data were strictly maintained throughout the research process.

## Results

 This study examined 108 patients who presented with dyspeptic symptoms at the Shahid Motahari Clinic in Shiraz and had a confirmed diagnosis of *H. pylori* infection. After excluding patients who did not meet the study criteria, statistical analysis was performed on the remaining cohort. The mean age of the participants was 43.92 ± 11.72 years, with 33 men (30.6%) and 75 women (69.4%).

 At baseline, the diagnostic performance of the three non-invasive methods—UBT, stool antigen test, and anti-*H. pylori* IgG serology was evaluated against the invasive gold standard (endoscopic biopsy with rapid urease test and histopathology). Among these, the UBT demonstrated the highest diagnostic accuracy, with a sensitivity of 100%, specificity of 94.6%, positive predictive value (PPV) of 96.8%, and negative predictive value (NPV) of 100%, confirming its reliability for initial *H. pylori* detection.

 Follow-up evaluation 1 month after therapy completion showed that UBT results converted to negative in 92% of patients, underscoring its strong utility for monitoring eradication. In the same period, stool antigen levels decreased significantly, with 78% of patients achieving negative conversion, confirming moderate usefulness for follow-up but with lower accuracy compared to UBT. Anti-*H. pylori* IgG serology also showed a reduction in titers; however, only 65% of patients demonstrated a meaningful decline, and persistent antibody positivity limited its role as a reliable indicator of eradication success.

 Thus, while all three non-invasive tests provided information on treatment response, UBT was the most accurate method for both initial diagnosis and confirming eradication, followed by stool antigen testing, whereas serology had limited post-treatment value due to delayed antibody decline.

 Further analysis explored whether diagnostic test results were associated with specific gastrointestinal complaints. No significant differences were found between the performance of UBT, stool antigen, or IgG serology across patients presenting with epigastric pain, reflux, heartburn, nausea, bloating, anorexia, constipation, or belching (*P* > 0.05 for all comparisons) (Table 1).

**Table 1:** Frequency of clinical symptoms in patients with *H. pylori* infection

**Symptom**	**Number**	**Percent**
Epigastric pain	81	75.0%
Reflux	18	16.7%
Heartburn	15	13.9%
Nausea	9	8.3%
Bloating	12	11.1%
Anorexia	9	8.3%
Constipation	15	13.9%
Belching	3	2.8%

 Patients were also asked how long their dyspepsia symptoms had lasted, with an average duration of 43.4 ± 31.14 months. We measured this duration from symptom onset to the start of drug treatment. At the second visit, 1.5 months after the first visit and one month after the end of drug treatment, 45 patients reported that their initial complaints had resolved and they were asymptomatic. Conversely, 63 patients still had symptoms and reported the same clinical complaints as at the first visit.

 The results of the stool antigen levels before and after treatment are illustrated in Figure 1. The mean stool antigen level before treatment was 4.2 ± 0.64, which significantly decreased to 2.9 ± 0.75 after treatment (*P* = 0.03). This significant reduction in stool antigen levels indicates the treatment’s effectiveness in reducing *H. pylori* infection (Figure 1).

**Figure 1:**
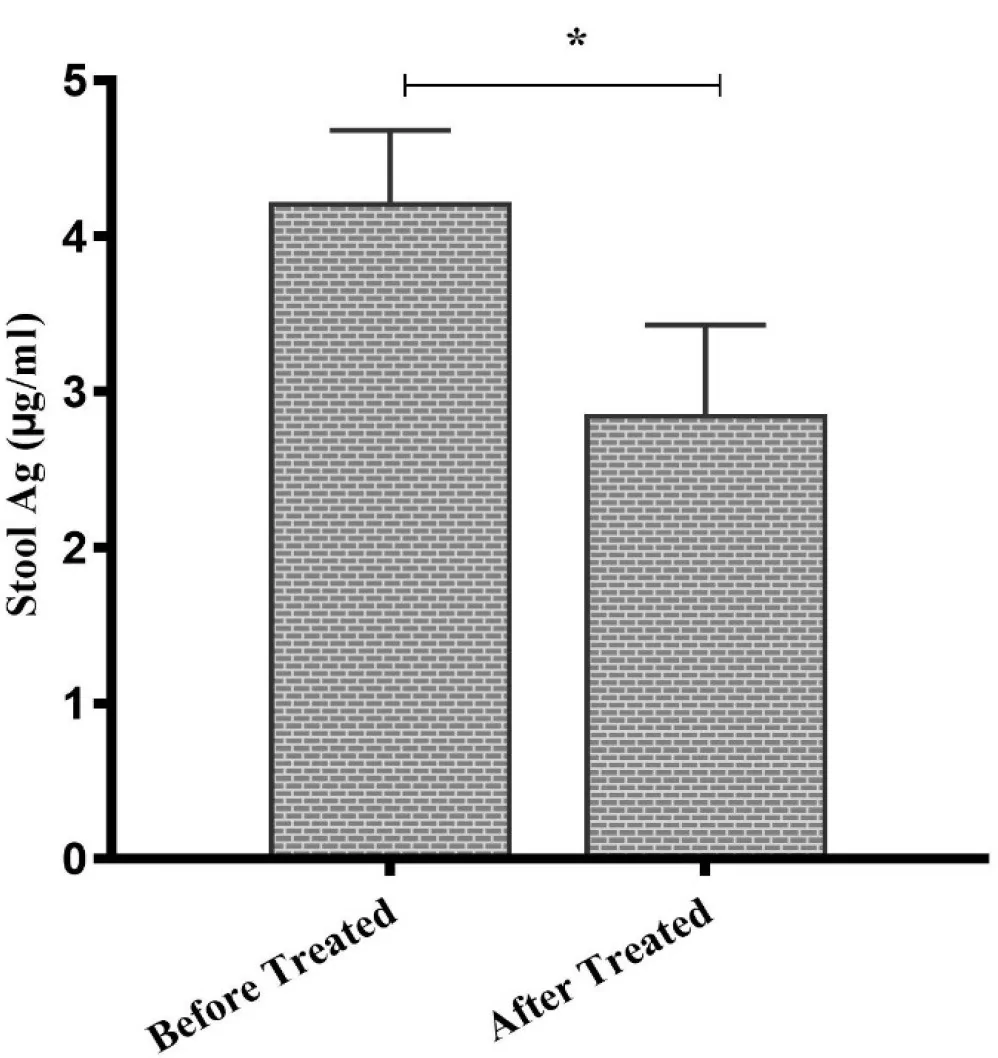
Comparison of stool antigen levels before and after treatment

 Between the two treated groups and those who did not complete the treatment, the variables “duration of symptoms”, “age”, and “sex” had no significant relationship with “decrease in fecal antigen level” (*P* < 0.05).

 On the other hand, although the fecal antigen level decreased by 4.3 in the treated group and increased by 1.3 in the untreated group, the difference was not statistically significant. Also, there was no significant difference in stool antigen changes between patients whose indigestion symptoms resolved after drug treatment and patients who remained symptomatic (*P* < 0.05). The comparison of changes in fecal antigen levels between the two treated and untreated groups is shown in Table 2.

**Table 2:** Comparison of changes in stool antigen levels with or without clinical symptoms

**Clinical Symptom**		**Stool Antigen Change ** **(Mean±SD)**	***P*** ** value**
Epigastric pain	Yes	-1.2 ± 0.7	0.82
	No	-1.6 ± 2	
Reflux	Yes	-0.1 ± 1.6	0.49
	No	-1.6 ± 0.8	
Heartburn	Yes	-2 ± 1.2	0.71
	No	-1.2 ± 0.8	
Nausea	Yes	-4.7 ± 3.2	0.18
	No	-1 ± 0.8	
Bloating	Yes	-6 ± 2.9	0.19
	No	-0.8 ± 0.7	
Anorexia	Yes	-3.5 ± 0.9	0.39
	No	-1.1 ± 0.8	
Constipation	Yes	-0.5 ± 3	0.67
	No	-1.5 ± 0.7	
Belching	Yes	0	0.77
	No	-1.4 ± 0.8	

 The results of the anti *H. pylori* IgG titers before and after treatment are shown in Figure 2. The mean IgG titer before treatment was 54.26 ± 3.33, which significantly decreased to 46.51 ± 3.22 after treatment (*P* ≤ 0.0001). This significant reduction in antibody titers indicates the treatment’s effectiveness in attenuating the immune response to *H. pylori* infection (Figure 2).

**Figure 2:**
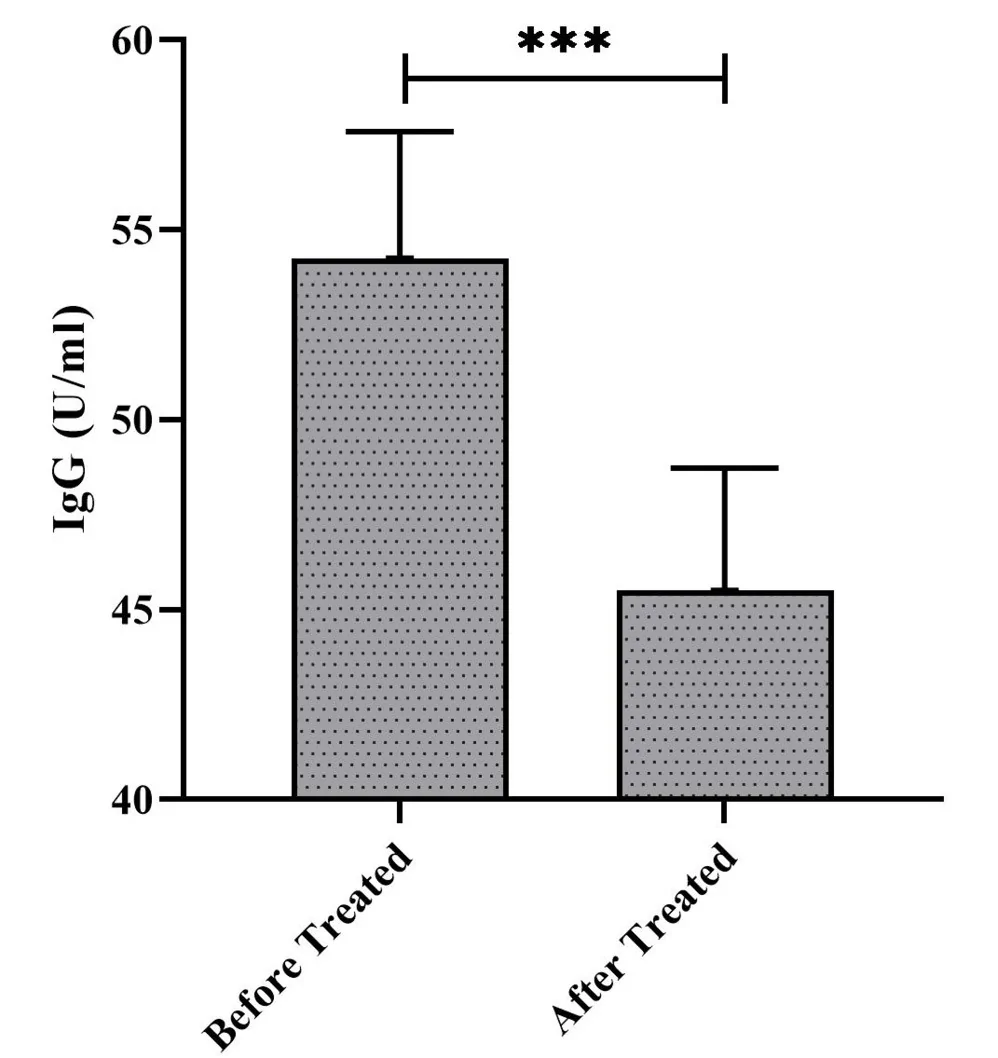
Comparison of anti *H. pylori* IgG titers before and after treatment

 There was no significant difference in antibody titer reduction between patients whose clinical symptoms resolved after drug treatment and those who remained symptomatic (*P* > 0.05). Also, the antibody titer decreased in the treated group, but this decrease did not show a significant association with the patients’ clinical symptoms (*P* < 0.05). The comparison of IgG antibody titers between the two groups, treated and untreated, is shown in Table 3.

**Table 3:** Comparison of changes anti *H. pylori* IgG levels with or without clinical symptoms

**Clinical symptom**		**IgG Change** ** (Mean±SD)**	***P*** ** value**
Epigastric pain	Yes	-6.9 ± 1.4	0.13
	No	-2.1 ± 3.1	
Reflux	Yes	-6.3 ± 2.2	0.85
	No	-5.6 ± 1.6	
Heartburn	Yes	-6.2 ± 7.8	0.95
	No	-5.7 ± 1.1	
Nausea	Yes	-5 ± 1.9	0.87
	No	-5.8 ± 1.5	
Bloating	Yes	-9.4 ± 3.1	0.36
	No	-5.3 ± 1.5	
Anorexia	Yes	-6.1 ± 2.7	0.93
	No	-5.7 ± 1.5	
Constipation	Yes	-11.6 ± 3.3	0.92
	No	-4.8 ± 15	
Belching	Yes	-2.3 ± 0	0.67
	No	-5.8 ± 1.4	

 Additionally, the relationship between initial and secondary antibody titers and age, sex, and duration of treatment was examined, and none were significant (*P* > 0.05).

## Discussion

 The present study evaluated different non-invasive diagnostic methods for *H. pylori* infection and their role in assessing treatment outcomes. Among the 108 patients studied, baseline analysis confirmed that UBT had the highest diagnostic accuracy compared with stool antigen testing and IgG serology. When these non-invasive methods were compared against the invasive gold standard (endoscopic biopsy with rapid urease test and histopathology), UBT most closely matched the accuracy of invasive testing, while stool antigen testing and IgG serology demonstrated lower specificity. This comparison highlights that although invasive methods remain the reference standard, UBT offers a reliable non-invasive alternative with comparable diagnostic value, particularly useful in patients for whom endoscopy is unnecessary.^18,19^

 Following therapy, both stool antigen levels and IgG titers decreased significantly, demonstrating their usefulness for monitoring treatment response, although their specificity was lower than that of UBT. Similar studies have reported that while stool antigen testing and serology can provide supportive evidence for eradication, UBT remains the most reliable non-invasive method for confirming treatment success.^20,21^

 In contrast, previous studies have often concluded that eradication of *H. pylori* infection leads to improvement in patients’ symptoms, including those with functional and non-ulcer dyspepsia, and therefore recommend treatment and eradication.^22-24^ However, in our study, the stool antigen test showed only moderate diagnostic accuracy compared with UBT, with lower sensitivity and specificity. While stool antigen testing is considered a practical and widely used non-invasive tool, especially for follow-up after therapy, our findings indicate that it is less reliable than UBT for initial diagnosis. This aligns with the results reported in the abstract, which found that stool antigen testing was moderately effective. The absence of a significant relationship between eradication and symptom improvement in our cohort may also be partly explained by the lack of endoscopic evaluation to rule out other causes of gastrointestinal complaints, such as peptic ulcers or hiatal hernia, which could not be assessed in this study.

 In this study, the stool antigen test, which has demonstrated acceptable diagnostic accuracy and can serve as a non-invasive method for infection detection and diagnosis, served as the criterion for *H. pylori* infection in this study. Reports indicate that the stool antigen test’s diagnostic accuracy surpasses that of the PCR test.^25^ We divided patients into successful and unsuccessful treatment groups based on the stool antigen test results post-treatment and made further comparisons accordingly. Previous studies have indicated that the stool antigen test post-treatment is reliable and can serve as a criterion for determining the success or failure of the treatment.^26^

 In comparing non-invasive diagnostic tools for *H. pylori* infection, the present study found that the UBT demonstrated the highest diagnostic accuracy, with a sensitivity of 100%, specificity of 94.6%, PPV of 96.8%, and NPV of 100%. Furthermore, follow-up UBT performed 1 month after therapy showed a marked reduction in positive cases, highlighting its usefulness not only for initial diagnosis but also for confirming eradication outcomes. These findings are consistent with previous research. For example, the Cochrane review underscored UBT’s superiority over serology and stool antigen tests, particularly because of its low false-negative rate, which is crucial for both symptomatic and asymptomatic patients.^27^ Similarly, Redéen and others reported that UBT’s diagnostic accuracy was comparable to invasive methods, making it a reliable choice when endoscopy is not required.^28^ A recent pilot study noted the potential of IgG serology as a screening tool because of its high sensitivity, yet UBT remained superior in overall diagnostic performance and patient comfort.^29^ Mounsey and Leonard also confirmed that UBT maintains the highest sensitivity and specificity, especially in patients without recent antibiotic or proton pump inhibitor use.^30^

 The study emphasized timely drug therapy discontinuation and a 30-day interval between the end of drug therapy and the post-treatment stool antigen test. The study excluded any patient who did not meet these criteria. Previous research has also stated that the stool antigen test at this time (30 days post-treatment) has acceptable accuracy and zero false negatives.^31^ Anti-acid drugs also eliminate the false-negative effect on the test at this time.^32^ Additionally, this study considered the stool antigen test as the standard and compared it with antibody tests and their parameters. Previous studies have also taken this approach due to the ease, non-invasiveness, and acceptable sensitivity and specificity of the stool antigen test.^15^ The Iranian Journal of Pathology published a study stating that the quantitative amount of *H. pylori*-related stool antigen did not correlate with sex, but it did correlate with age (*P* = 0.03).^14^ However, the present study found no correlation between the quantitative amount of antigen and age, sex, or other variables.

 Researchers have found a link between the amount of stool antigen and the severity of stomach inflammation. The amount of stool antigen has also been used to figure out how many bacteria are in the digestive tract.^32,33^ In the present study, the group that successfully eradicated the infection showed a greater reduction in stool antigen levels than the untreated group. This study suggests that there is no correlation between the severity of *H. pylori* infection and factors such as sex, age, type or duration of clinical symptoms, or the quantitative amount of stool antigen, assuming a relationship between the quantitative amount of stool antigen and the bacterial load.

 The present study observed a decrease in the mean titer of anti-*H. pylori* IgG with drug therapy. Additionally, the antibody titer against the bacterium significantly decreased in the group that successfully eradicated the infection. The study by Lee and colleagues found that the anti-*H. pylori* IgG titer decreases over time in treated individuals and increases in cases of reinfection. In other words, a longer duration of infection is associated with a greater decrease in the Anti-*H. pylori* IgG titer. The present study found that individuals with dyspeptic symptoms for a longer duration did not have lower antibody titers than those with shorter durations. If we assume that the initial *H. pylori* infection coincides with the onset of symptoms, we can argue that the present study contradicts the aforementioned study.^34^

 Many studies have investigated the sensitivity and specificity of serological tests, yielding various estimates of sensitivity, specificity, positive predictive value, and negative predictive value. The present study observed that all individuals with a positive diagnostic test indicating *H. pylori* infection had anti-*H. pylori* IgG titers within the positive range (above 10 U/mL). We measured anti-*H. pylori* IgG titers again at the end of treatment, after treating 51 patients and finding 57 still infected with H. pylori, with all results falling within the positive range. Most sources indicate that serological tests have acceptable sensitivity but low specificity, which aligns with the findings of the present study.^35,36^

## Strengths and Limitations

 Among the study population, there was very little willingness to undergo the stool test, and many patients were reluctant to do so. This was one of the reasons for the study sample’s dropout. Some patients had to continuously use PPIs due to reflux or heartburn, and because of the false-negative effect of this drug category on the stool antigen test, we had to exclude these individuals from the study. Additionally, a large number of patients experienced significant symptom improvement after drug therapy and had no motivation for follow-up or further examination, which also contributed to sample dropout.

 Despite these limitations, this study has several strengths. It directly compared two widely available non-invasive diagnostic methods, stool antigen testing and anti-*H. pylori* IgG serology, before and after treatment, allowing for a clearer understanding of their relative diagnostic value. The study also benefited from a well-defined patient cohort, a standardized treatment protocol, and the inclusion of follow-up testing after therapy, all of which strengthened the reliability and clinical relevance of the findings.

## Conclusion

 The results of the present study indicate a significant reduction in the specific IgG antibody titer against the bacteria following treatment. Also, there was a significant drop in the average level of H. pylori antigen in stool after treatment. This is because *H. pylori* antigen is a sign of how many bacteria are in the digestive tract. It is noteworthy that the antibody titer in the treated group, which had a negative stool antigen test post-treatment, was significantly lower than in the untreated group.

 Furthermore, this study concludes that the patient’s clinical symptom resolution does not necessarily indicate the eradication of the bacteria or the resolution of the infection. Furthermore, neither demographic characteristics nor clinical symptoms are associated with the antibody titer.
